# Frontiers and future of immunotherapy for pancreatic cancer: from molecular mechanisms to clinical application

**DOI:** 10.3389/fimmu.2024.1383978

**Published:** 2024-05-02

**Authors:** Rui Zheng, Xiaobin Liu, Yufu Zhang, Yongxian Liu, Yaping Wang, Shutong Guo, Xiaoyan Jin, Jing Zhang, Yuehong Guan, Yusi Liu

**Affiliations:** ^1^ Department of Medical Immunology, Medical College of Yan’an University, Yanan, Shaanxi, China; ^2^ Department of Hepatobiliary Surgery, The Affiliated Hospital of Yan’an University, Yan’an, Shaanxi, China

**Keywords:** immune therapy, immune checkpoint inhibitors, cancer vaccines, adoptive cell therapy, pancreatic cancer

## Abstract

Pancreatic cancer is a highly aggressive malignant tumor, that is becoming increasingly common in recent years. Despite advances in intensive treatment modalities including surgery, radiotherapy, biological therapy, and targeted therapy, the overall survival rate has not significantly improved in patients with pancreatic cancer. This may be attributed to the insidious onset, unknown pathophysiology, and poor prognosis of the disease. It is therefore essential to identify and develop more effective and safer treatments for pancreatic cancer. Tumor immunotherapy is the new and fourth pillar of anti-tumor therapy after surgery, radiotherapy, and chemotherapy. Significant progress has made in the use of immunotherapy for a wide variety of malignant tumors in recent years; a breakthrough has also been made in the treatment of pancreatic cancer. This review describes the advances in immune checkpoint inhibitors, cancer vaccines, adoptive cell therapy, oncolytic virus, and matrix-depletion therapies for the treatment of pancreatic cancer. At the same time, some new potential biomarkers and potential immunotherapy combinations for pancreatic cancer are discussed. The molecular mechanisms of various immunotherapies have also been elucidated, and their clinical applications have been highlighted. The current challenges associated with immunotherapy and proposed strategies that hold promise in overcoming these limitations have also been discussed, with the aim of offering new insights into immunotherapy for pancreatic cancer.

## 1 Introduction

Pancreatic cancer is a highly malignant digestive tract tumor, which has a poor prognosis and is associated with a higher risk of local invasion and distant metastasis (1). Although early surgical resection is the preferred treatment for these tumors (2), most patients are already in an advanced stage at diagnosis and less than 20% cases are suitable for surgical resection (3). The prognosis of patients with advanced disease is poor, with a 5-year survival rate of only 5% (4, 5). Pancreatic cancer includes various pathological types including ductal adenocarcinoma, acinar cell carcinoma, and small cell carcinoma, among others (6). Ductal adenocarcinoma is the most common histological type, and accounts for over 90% of all cases. As per new estimates, pancreatic ductal adenocarcinoma (PDAC) was projected to account for 3% of cancer incidence in 2023, with an estimated mortality rate of 8% (7). In this context, the mortality from pancreatic cancer has not decreased significantly owing to delayed diagnosis, early metastasis, and limited efficacy of chemotherapy or radiotherapy; systemic chemotherapy remains one of the main treatment options (8–10). Gemcitabine-based chemotherapy is currently the standard for metastatic pancreatic ductal adenocarcinoma (mPDAC) (11). Although combined gemcitabine and oxaliplatin, irinotecan, leucovorin, and fluorouracil have all been found to reduce patient mortality, and the modified FOLFIRINOX (5-fluorouracil, leucovorin, irinotecan, and oxaliplatin) regimen has slightly improved survival in these patients, the 5-year survival rate remains at approximately 8% (12, 13). It is therefore essential to identify more effective treatments for this condition.

**Figure 1:**
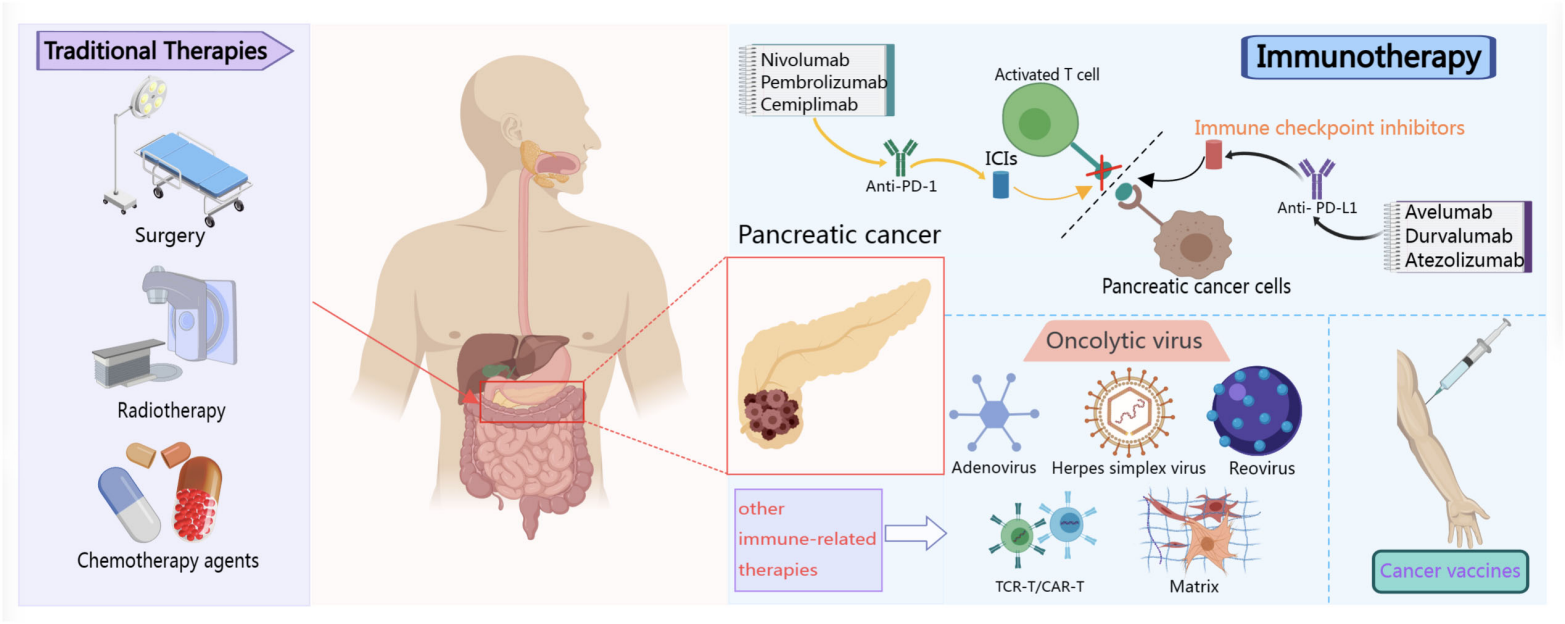
Schematic diagram of several methods commonly used in the clinical treatment of pancreatic cancer.

Surgery, radiotherapy, and chemotherapy are the traditional treatment options for pancreatic cancer (14). However, the tumors are usually beyond the scope of surgery in the advanced stages and the benefits from radiotherapy and chemotherapy are limited. Immunotherapy has increasingly gained attention for the treatment of pancreatic cancer owing to its specific effects on pancreatic cancer (15, 16). It is currently considered the fourth major modality for the treatment of these cancers (17), and is widely used in clinical practice in conjunction with traditional modalities (**Figure 1**). Reports indicate that tumor immunotherapy was first employed in the 1890s (18). William Coley was the first to administer an intratumoral injection of bacterial extracts to treat tumors. In 1967, Lindenmann and Klein (19) found that influenza virus-infected tumor cells could induce an anti-tumor response in host cells on inoculation into mice. Subsequent studies have increasingly confirmed the important role of the human immune system in the initiation and development of malignant tumors (20). Therefore, the role of immunotherapy in clinical practice has become increasingly prominent, and it has become an important breakthrough in the field of pancreatic cancer treatment. In addition, pancreatic cancer is often accompanied by immune escape and immunosuppression, and the effect of traditional treatment methods is limited (21). Immunotherapy is a treatment that activates the body’s immune system to fight tumors. It can kill tumor cells and control tumor development by stimulating and enhancing the patient’s immune response (22, 23). At present, drugs such as immune checkpoint inhibitors (ICIs) in pancreatic cancer immunotherapy have shown certain activity in clinical trials, and personalized immunotherapy strategies such as adoptive cell therapy and vaccine therapy are also being developed (24, 25). In addition, the field of pancreatic cancer immunotherapy is constantly exploring new potential biomarkers and immunotherapy combinations to improve the therapeutic effect. The in-depth research on the immune escape mechanism of pancreatic cancer and the development of targeted therapy will provide broader prospects for the application of immunotherapy in the treatment of pancreatic cancer.

Research indicates that pancreatic cancer has an unique immunosuppressive microenvironment (26), with extremely low T-cell infiltration and a relatively low mutation rate. These factors lead to successful immune tolerance in pancreatic cancer cells, and facilitate escape from the host immune system (27). Notably, the tumor microenvironment (TME) plays a key role in the occurrence, development, and metastasis of pancreatic cancer. This affects tumor growth and plays a key role in drug therapy and immune checkpoint inhibitors (ICIs) resistance (28). Coordination between various immune cells is necessary for achieving an effective anti-tumor immune response against pancreatic cancer (29, 30). These cells include cytotoxic CD8^+^ T cells, T helper cells, mature dendritic cells (DCs), macrophages, and natural killer (NK) cells, among others. However, various mechanisms have evolved to inhibit immune responses during the onset and development of pancreatic cancer. These result in the formation of an immunosuppressive microenvironment, which limits the activation and function of immune cells in the TME (31). This phenomenon has considerable impact on the clinical effects of immunotherapy and has become an important limiting factor for its success in pancreatic cancer. It is therefore essential to urgently identify new therapeutic methods or techniques for improving the prognosis of these patients.

**Figure 2:**
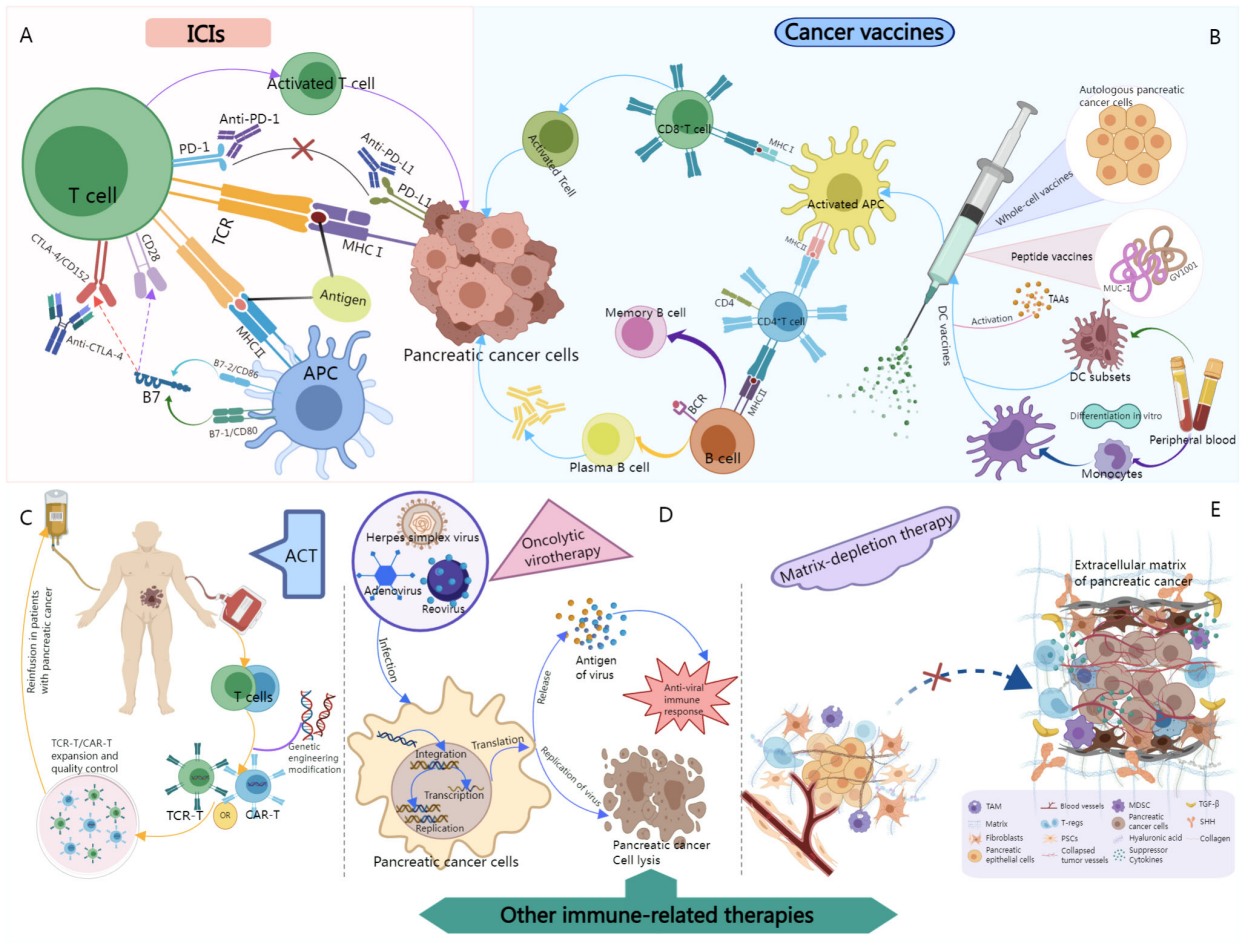
Schematic illustration of the molecular mechanisms of relevant immunotherapies for pancreatic cancer. **(A)** After binding to PD-1 or PD-L1, ICIs block the binding of tumor cells to T cells, thereby maintaining the tumor-killing activity of T cells and acting on pancreatic cancer. **(B)** Tumor antigens are delivered into patients in a variety of forms, such as autologous tumor cells, tumor-associated proteins or peptides, and dendritic cells as delivery vectors. After the vaccine enters the body, tumor antigens are phagocytosed by dendritic cells, which are then processed and presented to T cells to activate antigen-specific cytotoxic T cells, thereby killing pancreatic cancer. **(C)** The patient’s own immune cells were collected, genetically modified, and cultured *in vitro*, and then returned to the patient’s body to act on pancreatic cancer. **(D)** Oncolytic viruses replicate in tumor cells and can specifically infect and lyse tumor cells. In addition, the expression of viral antigens induces an antiviral immune response that can help destroy pancreatic cancer cells. 2E, Extracellular matrix-modified pancreatic cancer cells. Normally, pancreatic epithelial cells are surrounded by extracellular matrix (ECM), fibroblasts, and blood vessels to provide structural and nutritional support. However, during tumorigenesis, molecules such as tumor growth factors (such as TGF-β) or Hedgehog (SHH) activate intracellular signaling, leading cancer-associated fibroblasts to enhance ECM deposition. Forming a tumor microenvironment conducive to the growth and spread of pancreatic cancer. However, by blocking this pathway, it is possible to establish a more beneficial and healthy tumor microenvironment.

This review summarizes the various approaches commonly used in the treatment of pancreatic cancer in the clinic, and explores some new potential biomarkers and immunotherapy combinations. With particular emphasis on the molecular mechanisms of action of ICIs, cancer vaccines, adoptive cell therapy (ACT), oncolytic virotherapy, and matrix-depleting therapy (**Figure 2**). Findings from the latest in-depth studies on the clinical application of these therapies and recent progress made in the field have also been discussed. In addition, the current frontiers and future of immunotherapy in this cancer type have been described and strategies have been suggested to overcome existing limitations.

## 2 ICIs

**Figure 3:**
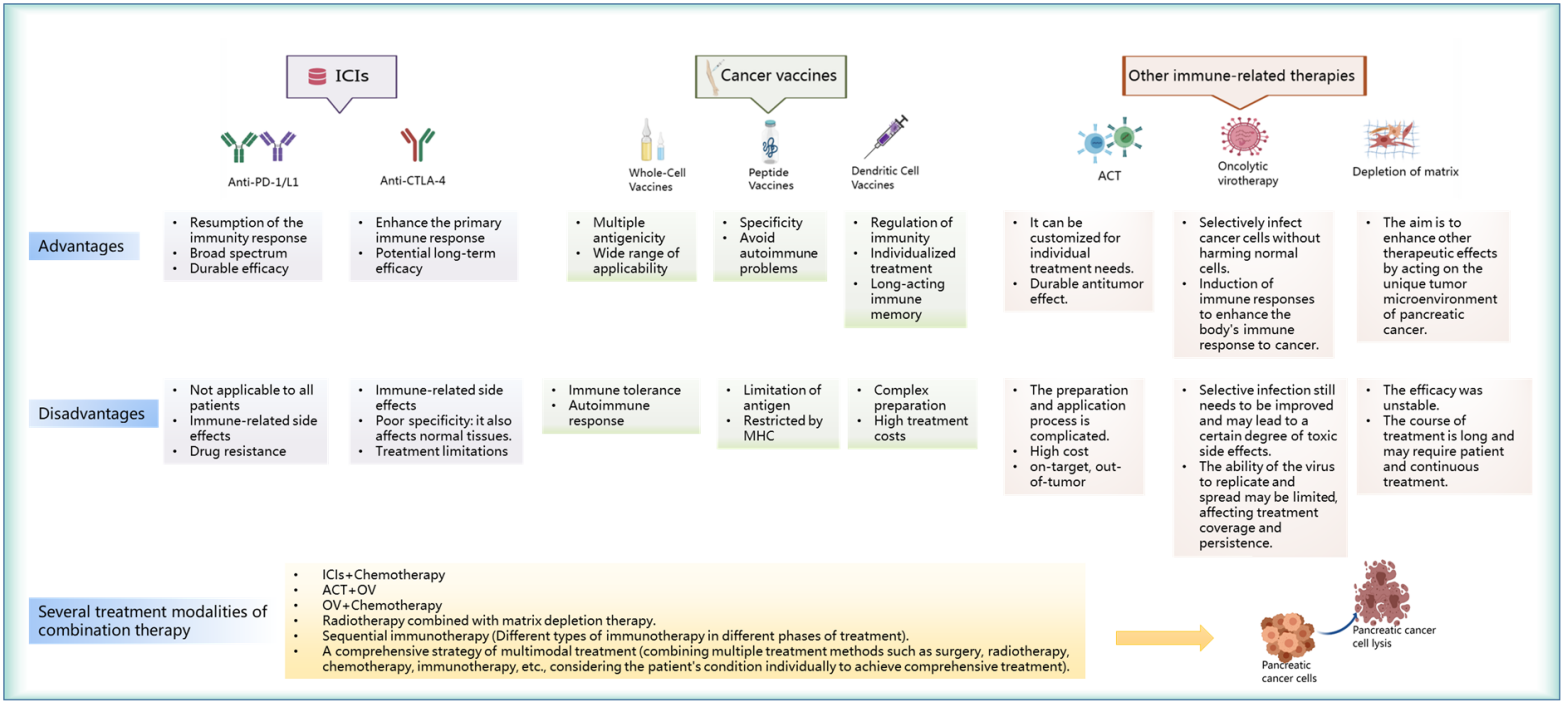
Advantages and disadvantages of immunotherapy for pancreatic cancer and the modes of action of combined immunotherapy in these cases.

ICIs represent a revolutionary form of cancer immunotherapy (32), that enhance anticancer immune responses by targeting immune receptors on the surface of T lymphocytes (33). The programmed death-1 (PD-1)/programmed cell death 1 ligand 1 (PD-L1) (34, 35) and cytotoxic T lymphocyte-associated antigen-4 (CTLA-4) immune checkpoints have gained considerable attention in the treatment of pancreatic cancer (36). PD-L1 is usually expressed by pancreatic cancer cells (37), and may potentially inhibit T-cell activity by binding to PD-1. Inhibition of the interaction between PD-1 and PD-L1 (38), or the inhibition of CTLA-4, can activate T cells (39) and enhance the immune attack on pancreatic cancer cells. The discovery of anti-PD-1/PD-L1 and anti-CTLA-4 has led to definite changes in the field of cancer immunotherapy (36). However, the use of these targets is also associated with various advantages and disadvantages (**Figure 3**). Anti-PD-1/PD-L1 can enhance the immune attack on cancer cells and is suitable for a variety of cancer types (40, 41); this ensures maintenance of long-term efficacy after drug withdrawal. However, this is associated with certain disadvantages and may lead to the development of side effects owing to possible triggering of the immune system (42), which may attack normal tissues and cause drug resistance in some cases. In the case of anti-CTLA-4, the initial immune response may be enhanced (43); this may prompt the immune system to attack cancer cells earlier and potentially lead to long-term efficacy. However, the efficacy of anti-CTLA-4 is relatively limited in some cancer types. This often necessitates the use of combination therapy (44, 45), which also has side effects. Therefore, although ICIs provide a new approach for the treatment of pancreatic cancer, it is essential to consider the patient condition to optimize treatment outcomes. Current research on ICIs mainly focuses on molecules such as anti-PD-1/anti-PD-L1 and anti-CTLA-4 agents (**Table 1**). The mechanism of action and clinical applications of each inhibitor have been discussed below.

**Table 1:** Preclinical studies and clinical trials based on ICIs in the treatment of pancreatic cancer.

Target	Agent	ClinicalTrials.Gov Identifier	Combinatorial Agent(s)	Phase	Endpoint	Status
PD-1	Nivolumab	NCT0324250	Sotigalimab(CD40 agonistic antibody), Gemcitabine/nab-paclitaxel	The randomized phase 2 PRINCE trial	OS, PFS, ORR, DCR	Completed
PD-1	Nivolumab	NCT02309177	Nab-Paclitaxel Plus Gemcitabine	Phase I	DLTs, OS, PFS	Terminated
PD-1	Pembrolizumab	/	Pelareorep(Oncolytic Reovirus); 5-Fluorouracil, Gemcitabine, Irinotecan	Phase Ib single-arm study	DLTs, Safety	Completed
PD-1	Pembrolizumab	NCT02704156	SBRT; Trametinib; Gemcitabine	Phase 2	OS	Completed
PD-L1	Avelumab	NCT03637491	Talazoparib; Binimetinib	Phase Ib of the JAVELIN PARP MEKi trial	DLTs	Completed
PD-L1	Durvalumab	NCT02879318	Tremelimumab; Gemcitabine; Nab-Paclitaxel	The CCTG PA.7 phase II trial	OS, PFS, ORR	Completed
PD-L1	Atezolizumab	NCT03193190 NCT03281369	PEGPH20; Chemotherapy	MORPHEUS Phase Ib/II	ORR, Safety	Completed
CTLA-4	Ipilimumab	/	Gemcitabine	Phase Ib	MTD, OS, PFS, OS,	Completed
CTLA-4	Ipilimumab	/	GVAX	Phase II	OS	Suspended
CTLA-4	Tremelimumab	NCT00556023	Tremelimumab (CP-675,206); Gemcitabine	Phase I	DLTs	Completed

Overall survival (OS), progression-free survival (PFS), objective response rate (ORR), disease control rate (DCR), dose-limiting toxicities (DLTs), stereotactic body radiotherapy (SBRT), Canadian Cancer Trials Group (CCTG), PEGylated recombinant human hyaluronidase (PEGPH20), Allogeneic GM-CSF-Transfected Pancreatic Tumor Vaccine (GVAX), Tremelimumab (CP-675,206) (a fully human monoclonal antibody).

### 2.1 Anti-PD-1 and anti-PD-L1 inhibitors

PD-1 is an inhibitory receptor for T cells, which is mainly expressed on the surface of activated T, B, and NK cells (46). PD-1 has two ligands, namely, PD-L1 and PD-L2 (47), of which PD-L1 is the major ligand. Notably, PD-L1 is expressed by different cell types including those of many different tumors (48). The combination of PD-1 and PD-L1 also inhibits T cell proliferation and reduces T cell survival; this allows tumor cells to evade immune surveillance (49). In this context, the PD-L1 gene is a proto-oncogene that is involved in the occurrence and development of pancreatic cancer. It is upregulated in this condition and is associated with tumor progression and poor prognosis (50, 51). PD-1/PD-L1 inhibitors are currently approved by the United States Food and Drug Administration (FDA) for the clinical treatment of a variety of malignant tumors (52).

The PD-1 inhibitors mainly include nivolumab, pembrolizumab, and cemiplimab, among others (53, 54). Studies on the treatment of pancreatic cancer have found that the efficacy of PD-1/PD-L1 inhibitors may be improved by combining with other therapies. In this context, an efficacy analysis of data (55) from 105 patients (included in a randomized phase II trial that evaluated sotigalimab and/or nivolumab plus chemotherapy in first-line metastatic pancreatic cancer) showed that the primary endpoint of 1-year overall survival (OS) was met in the nivolumab/chemotherapy arm (57.7%, P = 0.006). The historical 1-year OS was 35% (n = 34); this indicated that nivolumab combination therapy is effective in metastatic pancreatic cancer. Another phase I trial (56) that evaluated outcomes with nivolumab in combination with nab-paclitaxel and gemcitabine in 98 patients with locally advanced or metastatic pancreatic cancer, found the median progression-free survival (PFS) and OS to be 5.5 and 9.9 months, respectively. This indicated that the combination was safe in advanced pancreatic cancer and the adverse events were manageable. For the treatment of refractory metastatic pancreatic cancer, one study looked at 84 patients (57), of whom 41 received stereotactic body radiation therapy (SBRT) with nivolumab and 43 received SBRT with nivolumab and ipilimumab. The results showed that 17.1%(8.0 to 30.6) of the patients who received SBRT plus nivolumab had a benefit. In contrast, 37.2%(24.0 to 52.1) of patients who received SBRT with nivolumab and ipilimumab had a benefit. One patient who received SBRT plus nivolumab had a 4.6 month partial response. Overall, this study confirms that SBRT combined with nivolumab and ipilimumab has potential value in terms of antitumor activity and safety. However, the specific mechanism of SBRT is still unclear, and further in-depth studies are needed. Humanized IgG1 monoclonal antibodies (represented by pembrolizumab) play an important role in disrupting the PD-1/PD-L1 pathway (58). Based on the robust objective responses and excellent pharmacokinetics and safety data, pembrolizumab has received FDA approval for the treatment of various tumor types (59), including pancreatic cancer (60). However, pembrolizumab is applicable to only a minority of patients with pancreatic cancer, because not all patients have tumors that express PD-L1, and the heterogeneity of pancreatic cancer limits its efficacy (61). This indicates that the individual condition of the patient and the characteristics of the tumor should be considered comprehensively in the treatment selection.

Notably, pelareorep (62), an intravenously delivered oncolytic reovirus, is known to induce a T-cell inflammatory phenotype in PDAC. A phase Ib study (63) that evaluated pembrolizumab combined with pelareorep and chemotherapy included 11 patients with advanced pancreatic cancer. Efficacy of the combination was demonstrated in 10 patients, among whom 3 experienced disease control. One patient demonstrated partial response for up to 17.4 months; the 2 other patients had stable disease for 9 and 4 months, respectively. These results demonstrated that the addition of pelareorep and pembrolizumab to chemotherapy offered good efficacy without any significant increase in toxicity. However, another open-label randomized controlled phase 2 trial (64) compared SBRT plus pembrolizumab and trametinib with SBRT plus gemcitabine for locally recurrent pancreatic cancer (after surgical resection). Among 198 patients who underwent screening, 170 were enrolled and randomly assigned to receive SBRT plus either pembrolizumab and trametinib or SBRT plus gemcitabine. Serious adverse events were reported in both the SBRT plus pembrolizumab and trametinib group and the SBRT plus gemcitabine group. However, no treatment-related deaths were observed. These results suggest that the combination of SBRT with pembrolizumab and trametinib may represent a novel treatment option for patients with locally recurrent pancreatic cancer following surgery. Although a phase 3 trial is needed for further validation, this study offers promising directions for future treatment. In this context, the FDA approved another PD-1 inhibitor, namely, cemiplimab-rwlc (Libtayo), on February 22, 2021 for the first-line treatment of advanced non-small cell lung cancer (65) with high PD-L1 expression (tumor proportion score ≥ 50%); it was approved for either metastatic or locally advanced cases without *EGFR*, *ALK*, or *ROS1* mutations. However, a few clinical trials have evaluated the use of cemiplimab for the treatment of pancreatic cancer (66). There is a need for further in-depth research in this area.

PD-L1 inhibitors prevent tumor cells from being disguised as normal cells, and thereby facilitate their killing by T cells (67, 68). In this context, most PD-L1 inhibitors are IgG1 antibodies. IgG1 facilitates recognition of pathogenic antigens and can recognize PD-L1 expressed on the surface of tumor cells (69). The well-known PD-L1 inhibitors include avelumab, durvalumab, and atezolizumab (54, 70). Numerous clinical studies have found that although some tumors show high PD-L1 expression (71), PD-L1 monoclonal antibodies do not offer optimal therapeutic effect. A more comprehensive treatment regimen has therefore been explored in a phase Ib trial (72). Trials on avelumab or talazoparib plus binimetinib in mPDAC have shown promising additive or synergistic antitumor activity. Durvalumab is another humanized IgG1 monoclonal antibody (73) that targets PD-L1 and activates anti-tumor immunity by blocking the interaction between PD-L1 and its receptor, PD-1. It was first approved by the FDA in 2017 for the treatment of locally advanced or metastatic urothelial carcinoma (74) and stage III unresectable non-small cell lung cancer (75). In this context, a phase II trial (76) that employed gemcitabine and nab-paclitaxel with or without durvalumab and tremelimumab in mPDAC showed limited immunological efficacy of these agents. The scope of single-agent immunotherapies is also limited to mismatch-repair deficiencies. These findings suggest that the use of dual checkpoint inhibitors in combination with chemotherapy may be safe and effective in a specific range of tumors.

The IgG1 monoclonal antibody, atezolizumab, was designed with Fc domain modification to reduce antibody-mediated cytotoxicity and thereby prevent PD-L1-expressing T-cell depletion. Although research on the use of atezolizumab for pancreatic cancer is limited, the agent has shown promising efficacy in other tumor types including, metastatic breast cancer (77), and advanced alveolar soft part sarcoma (78). The positive outcomes in these tumors have considerably increased interest in its potential use for pancreatic cancer. More relevant studies are needed in the future to understand the potential effects of atezolizumab in the treatment of pancreatic cancer.

### 2.2 CTLA-4 inhibitors

CTLA-4 (CD152) plays an important role in the human immune system and is mainly expressed by activated CD4^+^/CD8^+^ T cells and CD25^+^/FOXP3^+^ regulatory T cells. Although CTLA-4 is homologous to CD28, it can interact with B7-1/B7-2 with higher affinity. In addition, it can regulate or even inhibit CD28 signal transduction, and thereby inhibit the activity of effector T cells (79–81). It can also reduce the production of interleukin-2, which may activate regulatory T cells and thereby weaken the immune response; this may in turn inhibit an anti-tumor immune response (82). The CTLA-4 inhibitors currently in common use include ipilimumab and tremelimumab (83), both of which have been tested in clinical trials. The data from clinical trials on these agents have been described below.

Ipilimumab treatment of melanoma prolongs patients’ survival by 4 months (84). In 2011, the FDA approved ipilimumab for the treatment of advanced melanoma (85), which opened a new era of immunotherapy in cancer treatment.

In the context of pancreatic cancer, a phase Ib study (86) that included 21 patients had evaluated the use of ipilimumab plus gemcitabine for the treatment of advanced PDAC, 3, 10, and 8 patients demonstrated partial response, stable disease, and disease progression, respectively. The objective response rate was found to be 14% (3/21) and the median PFS and OS were 2.78 and 6.9 months, respectively. These results further suggest that the use of gemcitabine and ipilimumab is both safe and feasible in advanced pancreatic cancer. However, it is worth noting that adding ipilimumab to gemcitabine does not appear to be more effective than gemcitabine alone in these cases. In this context, a phase II study (87) that tested the efficacy of granulocyte-macrophage colony-stimulating factor (GM-CSF)-allogeneic pancreatic tumor cells (GVAX) and ipilimumab in mPDAC found that maintenance treatment with the combination did not improve OS after continuation of chemotherapy; in addition, the results were numerically worse in mPDAC. However, both clinical responses and biological effects (on immune cells) were observed. It is therefore necessary to further investigate new combinations of maintenance therapy for mPDAC. Overall, combination therapy warrants further investigation in pancreatic cancer.

Tremelimumab, another CTLA-4 inhibitor, is a fully humanized IgG2 monoclonal antibody against CTLA-4. Studies have reported on its anti-tumor effects in malignant tumors including melanoma (88), liver cancer (89), and colorectal cancer (90). In a phase Ib trial (91), tremelimumab combined with gemcitabine showed some success in the treatment of metastatic pancreatic cancer. Among the 34 included patients, 2 demonstrated partial response and a median OS of 7.4 months. These results demonstrate the safety and tolerability of this combination and warrant further study in patients with metastatic pancreatic cancer. In this context, the use of combination therapy is currently preferred because of the immunosuppressive microenvironment of pancreatic cancer, which makes it often difficult to obtain optimal efficacy with CTLA-4 inhibitors alone. The future direction of treatment requires the combination of chemotherapy and radiotherapy with immunotherapy in order to achieve better therapeutic effects.

Recently, unique barriers to the effectiveness of ICIs in pancreatic cancer include the following: First, PD-1/PD-L1 inhibitors are not suitable for all patients, as some have poor responses to them. In addition, the use of these drugs may trigger immune-related side effects, causing the immune system to attack normal tissues, and the problem of drug resistance may arise. Similarly, CTLA-4 inhibitors also trigger immune-related side effects that may cause the immune system to attack normal tissues. In addition, due to its poor specificity, it may have adverse effects on normal tissues. The efficacy of this treatment in some types of cancer is relatively limited, and it often needs to be used in combination with other treatments.

## 3 Cancer vaccines

Over the past decade, considerable progress has been made in the use of cancer vaccines for solid tumors (25). They represent one of the most widely studied types of tumor immunotherapy, especially for pancreatic cancer (61, 92). In this context, development of the tumor leads to a gradual weakening of the monitoring and recognition ability of the immune system; this allows tumor cells to escape immune clearance. However, tumor vaccines can stimulate the expansion, amplification, or activation of tumor-specific T/B cells via active immunization (93), and thereby enhance the anti-immune response to tumors; this facilitates specific killing of tumor cells.

Vaccine-based anticancer immunotherapy aims to harness the ability to recognize and respond effectively to novel antigens (94). Therapeutic vaccines for pancreatic cancer currently include whole-cell, peptide, and DC vaccines. Each of the three vaccines have unique advantages and limitations (**Figure 3**). Whole-cell vaccines are suitable for a wide variety of tumors and have multi-antigenicity, but their use is limited by the issues of immune tolerance and autoimmune reactions (95). In contrast, peptide vaccines are specific and reduce the risk of autoimmune reactions (96–98); however, their use is limited by antigen- and major histocompatibility complex (MHC)-related constraints. DC vaccines can effectively stimulate and regulate the immune system (99) and may be potentially used for individualized treatment; however, their preparation involves a complex process and is expensive. As the field of immunotherapy continues to advance, clinical studies on these vaccines will provide key insights into the treatment of pancreatic cancer (**Table 2**). The mechanism of action of these three advanced vaccine types and their clinical application have been described in the subsequent sections.

**Table 2:** Preclinical studies and clinical trials based on cancer vaccines for the treatment of pancreatic cancer.

Type of immunotherapy	ClinicalTrials. Gov Identifier	Combinatorial Agent(s)	Phase	Endpoint	Status
Whole-Cell Vaccines	NCT02243371	GVAX pancreas vaccines; Cy; CRS-207; Nivolumab	/	OS, PFS	Completed
Whole-Cell Vaccines	NCT01072981	Algenpantucel-L	Phase 2	DFS, OS	Active
Whole-Cell Vaccines	NCT01836432	Algenpantucel-L, FOLFIRINOX, Gemcitabine/nab-paclitaxel	Phase 3	OS	Terminated
Peptide Vaccines	/	GM-CSF, Gemcitabine, GV1001	/	DTH, Proliferation, ELISPOT	Completed
Peptide Vaccines	ISRCTN4382138	Gemcitabine, Capecitabine, GV1001	Phase 3	OS	Completed
Peptide Vaccines	/	MUC-1	Phase 1	MOS,	Completed
Peptide Vaccines	/	MUC-1,WT1, Chemotherapy	Phase I/II	MOS,OS	Completed
Peptide Vaccines	NCT02261714	TG01/GM-CSF, Gemcitabine	Phase 1/2	OS,DFS	Completed
DC vaccines	/	LAK, Gemcitabine, S-1, DC vaccine	/	OS	Completed
DC vaccines	NCT01410968	Poly-ICLC, DC	/	Safety, Feasibility	Completed
DC vaccines	NCT01781520	DC-CIK, (CT) S-1	Prospective Study	OS, PFS	Completed

Disease-free survival (DFS), GM-CSF-secreting allogeneic pancreatic tumor cells (GVAX pancreas vaccine), cyclophosphamide (Cy), live, attenuated Listeria monocytogenes-expressing mesothelin (CRS-207), NewLink Genetics Corporation, Ames, IA (algenpantucel-L), disease-free survival (DFS), neoadjuvant SOC chemotherapy (FOLFIRINOX or gemcitabine/nab-paclitaxel),16-amino acid telomerase peptide (GV1001), Delayed-type hypersensitivity (DTH), Enzyme-Linked Immunospot Assay (ELISPOT), mucin-1 (MUC-1), median overall survival (MOS), Wilms' tumor 1 (WT1), cancer immunotherapy (TG01), lymphokine-activated killer (LAK), toll-like receptor (TLR)-3 agonist (Poly-ICLC), dendritic cells and cytokine-induced killer cells (DC-CIK).

### 3.1 Whole-cell vaccines

Whole-cell vaccines prime the patient immune system to attack pancreatic cancer cells. The process involves the presentation of cancer cell antigens by antigen-presenting cells and the activation of CD8^+^ T cells that directly kill the cancer cells; the CD4^+^ T cells provide helper signals. Vaccines are prepared using homologous cells or engineered cell lines and are combined with immune adjuvants to improve efficacy (100–103). This approach facilitates the removal of cancer cells and the formation of immune memory. In a preclinical study, high levels of expression of GM-CSF was found to stimulate durable antitumor activity with vaccine-based therapy (104). The GVAX vaccine was therefore developed for treating pancreatic cancer. It is an allogeneic, irradiated, whole-cell-based tumor vaccine which includes two pancreatic cancer cell lines that were engineered to express GM-CSF and irradiated to prevent further cell division. The vaccine was developed with the aim of stimulating a comprehensive immune response and providing a wider range of immunotherapy options for patients with pancreatic cancer (105). In the early 1990s, the allogeneic GVAX tumor vaccine was found to demonstrate a favorable safety profile in a phase I study (106) on PDAC. Three patients who received ≥10 × 10^7^ vaccine cells showed increased delayed hypersensitivity to autologous tumor cells. Based on these promising results, a trial (107) evaluated the use of cyclophosphamide (Cy)/GVAX+CRS-207 (live attenuated *Listeria monocytogenes* mesothelin expressing cells) with or without nivolumab. Although the combination did not significantly improve OS, an objective response was observed and the OS remained comparable to that obtained with existing therapies. In addition, significant immunological changes were induced in the TME. Further research is therefore warranted in this regard, and related combined treatment strategies need to be explored to improve the therapeutic effect.

Algenpantucel-L is another hyperacute rejection-based allogeneic pancreatic cancer vaccine, which consists of two PDAC cell lines (HAPa-1 and HAPa-2) that are genetically engineered to express α-galactose epitopes on membrane glycoproteins and glycolides (108); these lead to hyperacute rejection and thereby kill tumor cells. In a phase II trial (109) that evaluated algenpantucel-L plus gemcitabine or 5-fluorouracil-based chemoradiotherapy in patients with resected pancreatic cancer, the addition of algenpantucel-L improved survival compared with standard adjuvant therapy, offering a median PFS of 62% and OS of 86% at 12 months. This compares with the rates of 45% and 65%, respectively, that were reported in a previous study. These results further suggest that in combination with chemotherapy, algenpantucel-L is effective in treating patients with pancreatic cancer. In order to further explore the mechanism of action, another randomized clinical trial (110) included patients with PDAC. The patients were assigned to receive either standard chemoradiotherapy or standard neoadjuvant therapy plus algenpantucel-L (HyperAcute-Pancreas) immunotherapy. The results showed that 75% of patients in the standard group experienced adverse events of grade 3 or higher, while 81% of patients in the experimental group experienced similar adverse events; however, no significant difference was observed between the two groups. Although algenpantucel-L did not achieve the expected outcomes in this trial, its potential efficacy as a new option in the field of pancreatic cancer treatment warrants further investigation. Future trials may help to further evaluate its actual impact and potential role in the treatment of pancreatic cancer, and thereby provide more treatment options for affected patients.

### 3.2 Peptide vaccines

Peptide vaccines contain specific antigen fragments (111) directed against surface antigens of pancreatic cancer cells (112, 113). These antigen fragments may be presented to T cells by antigen-presenting cells; the subsequent activation of CD8^+^ T cells induces them to attack cancer cells. The activated T cells then proliferate to form more effector T cells that are capable of recognizing cancer cells (111). Finally, peptide vaccines help establish immune memory and provide patients with long-term protection against pancreatic cancer (114, 115). The involved process allows the patient immune system to mount a long-lasting immune response that is vigilant for the presence of pancreatic cancer.

Another cancer vaccine, namely, GV1001, is currently under clinical investigation for telomerase overexpression in pancreatic cancer; the vaccine contains a telomerase-derived peptide of 16 amino acids (116). In this context, a study (117) had used GV1001 in combination with GM-CSF (an immunostimulant) and gemcitabine as first-line therapy to investigate its safety and immunogenicity in patients with unresectable pancreatic cancer. The preliminary results indicated that GV1001 performed well in terms of safety when combined with chemotherapy, and patients developed telomerase-specific immune responses. These findings support the use of GV1001 as a potential therapeutic strategy, especially in patients with unresectable pancreatic cancer. However, in another important phase 3 trial (118) (where patients with locally advanced or metastatic pancreatic cancer were administered combined gemcitabine and capecitabine), the addition of GV1001 offered no significant improvement in OS compared with chemotherapy alone. This suggests that new strategies are needed to enhance the immune response to telomerase vaccines during chemotherapy, as this may improve clinical efficacy.

Mucin-1 (MUC-1) is a type I transmembrane protein that promotes tumor invasion, angiogenesis, and metastasis (119, 120). Notably, MUC-1 expression levels have been reported to be elevated in more than 60% of patients with pancreatic cancer, and the levels have been found to significantly correlate with tumor size (121). In addition, high MUC-1 expression is associated with poor prognosis in pancreatic cancer (122). In one of the therapeutic strategies against MUC-1, a vaccine was developed using a specific peptide to stimulate the patient’s own immune system against cancer cells. An initial study suggested MUC-1 peptide therapy to be relatively safe in patients with pancreatic cancer; it also suggested that MUC-1 helps to enhance the immune response to tumor antigens (123). In another initial clinical trial (124) that included patients with advanced pancreatic cancer, MUC-1 showed antigen-specific T-cell responses in 5 of 8 patients (62.5%) who could be evaluated. Notably, patients who developed vaccine-specific T-cell responses demonstrated significantly improved OS (15.1 and 3.9 months, respectively). These findings further elucidate the potential role of vaccine therapy in patients with pancreatic cancer.

Notably, a precise assessment of baseline antitumor-specific immunity is critical to the prediction of clinical outcomes. In a phase I/II study (125) that included 48 patients with pancreatic cancer, outcomes were predicted based on baseline immunity; the patients received Wilms tumor 1 and/or MUC-1 peptide-loaded DC vaccination and standard chemotherapy. No serious adverse events were observed in relation to vaccination, and the median PFS and OS were 8.1 and 15.1 months, respectively; in addition, the DC vaccine was found to have enhanced tumor-specific immunity. These results suggest that DC-based immunotherapy combined with conventional chemotherapy is safe and clinically beneficial for patients with advanced PDAC. This may represent a promising new strategy for the treatment of patients with recurrent or refractory PDAC with favorable prognostic factors.

### 3.3 DC vaccines

DCs play a key role in the immunotherapy of pancreatic cancer (126, 127). As professional antigen-presenting cells, DCs are essential for the T-cell response. They present extracellular antigens to CD4^+^ T cells via MHC class II molecules and intracellular antigens to CD8^+^ T cells via MHC class I molecules, demonstrating the phenomenon of cross-presentation (128, 129). DC vaccines are developed by loading tumor-associated antigens *in vitro (* 130) and then administering them to the patient to stimulate specific immune responses against pancreatic cancer (127, 131).

However, DC vaccines alone do not offer optimal efficacy in the treatment of pancreatic cancer due to the unique immunosuppressive microenvironment of pancreatic cancer (132). Nevertheless, relatively satisfactory results have been achieved when combined with other modalities such as radiotherapy and chemotherapy (133). Certain clinical trials have confirmed the safety and efficacy of DC vaccines in the treatment of pancreatic cancer (127). In this context, a clinical study (134) employed DC vaccines (alone or in combination with lymphokine-activated killer cells) along with gemcitabine and/or S1 in patients with inoperable pancreatic cancer. The results showed that DC vaccine combined with chemotherapy showed certain safety and efficacy for patients with advanced cancer refractory to standard therapy. However, the results of 2 complete responses, 5 partial responses, and 10 stable disease apply to a small proportion of the patients in the trial. In addition, the median survival was 360 days, but it should be noted that this is only a statistical measure and does not represent the prognosis of all patients. Therefore, further studies are needed to verify the reliability and applicability of these results.

Researchers have also explored the possibility of combining DC-based immunotherapy with polyinosinic-polycytidylic acid stabilized using polylysine and carboxymethylcellulose (a Toll-like receptor-3 agonist) for the treatment of pancreatic cancer (135). Autologous DCs derived from the peripheral blood of human leukocyte antigen-A2 patients were infused, and the agonist was administered on the day of vaccination. The findings suggested that the 12 participants showed good tolerance and the vaccine was successfully generated. On imaging at day 56, 4 of the 8 patients had stable disease and progressive disease, respectively, with a median OS of 7.7 months. In addition, MHC-I tetramer analysis demonstrated the successful formation of antigen-specific T cells in three patients with stable disease. This study provides a glimmer of hope for the treatment of pancreatic cancer.

These vaccines can overcome the immunosuppressive tumor microenvironment of pancreatic cancer, activate a variety of immune cells, break the immune tolerance state, enable the immune system to recognize and attack pancreatic cancer cells, promote the immune system to form long-term immune memory, and improve the long-term immune protection effect. In addition, the antigens provided by the vaccine can be effectively recognized and processed by the immune system, which further enhances the ability of the immune system to recognize and attack tumors. However, due to the unique immunosuppressive microenvironment of pancreatic cancer, it is difficult to use these tumor vaccines alone to achieve ideal effects. Therefore, the commonly used combination immunotherapy could improve the therapeutic effect in clinical practice, such as whole-cell vaccine combined with chemotherapy drugs such as gemcitabine, or combined with nivolumab. Peptide vaccine combined with chemotherapy drugs; dendritic vaccines combined with lymphokine-activated killer cells, gemcitabine and/or S-1, and DCs immunotherapy combined with Toll-like receptor (TLR)-3 agonists, etc. In order to fully exert the potential efficacy of tumor vaccines, it is necessary to design a multi-antigen combination vaccine that covers a variety of tumor-specific antigens and improves the broad spectrum of the vaccine. At the same time, it is necessary to take the unique characteristics of the immunosuppressive microenvironment of pancreatic cancer patients into account to enhance the adaptability of vaccines to this environment and adopt individualized strategies to consider the differences between different individuals. This comprehensive design is expected to provide a more effective immunotherapy for pancreatic cancer treatment.

Overall, the tumor vaccine trials enrolled small samples; larger sample sizes are needed to evaluate the efficacy of the vaccines in detail (136). In this context, patients with pancreatic cancer demonstrate a small number of effector T and NK cells in the peripheral blood; in addition, the TME inhibits the activity of effector T cells, thereby reducing the efficacy of the tumor vaccines (132, 137). New mechanisms of action need to be explored in order to fully exploit the potential efficacy of tumor vaccines, and multi-antigen combination vaccines (138) covering a wide variety of tumor-specific antigens need to be designed to broaden the spectrum of the vaccine. It is also necessary to consider the unique characteristics of the immunosuppressive microenvironment of pancreatic cancer, enhance vaccine adaptability to this environment, and adopt individualized strategies (139) based on individual differences. Vaccine designs based on this comprehensive approach may provide a more effective immunotherapy strategy for pancreatic cancer.

## 4 Other immune-related therapies

As a prominent research direction in the field of pancreatic cancer treatment, immunotherapeutic approaches have included various innovative strategies (140, 141). Among these, ACT (142), oncolytic virus therapy (143), and matrix depletion therapy (144) have shown significant therapeutic potential. Although they enhance functioning of the immune system to combat pancreatic cancer via different mechanisms, each therapeutic strategy has its own unique advantages and disadvantages (**Figure 3**). ACT offers the advantages of individualized treatment and durable antitumor effects, but is associated with the disadvantages of complex preparation methods and high costs (145, 146). Oncolytic viruses (OVs) offer the advantage of selectively infecting cancer cells and inducing immune responses; however, the process of selective infection needs to be improved and the replication ability is limited (147). The advantage of matrix depletion therapy lies in its ability to act on the unique TME of pancreatic cancer; it thereby enhances the effect of other treatments. However, it is associated with the disadvantages of an unstable effect and a long course of treatment. In summary, although these therapies bring new hope and offer more possibilities for the treatment of pancreatic cancer, further improvement and research are needed (148, 149). The subsequent sections provide a detailed description of the application of three immunotherapy modalities in the treatment of pancreatic cancer.

### 4.1 ACT

ACT involves the expansion and/or reactivation of autologous or allogeneic immune effector cells *in vitro* to increase immunogenicity and immunoreactivity. Rapid developments in the field of antigen identification, gene therapy, and T cell biology have led to the emergence of chimeric antigen receptor T (CAR-T) cell therapy and T cell receptor-engineered T cell (TCR-T) therapy (150, 151). Notably, the processes involved in the use of TCR-T and CAR-T for the treatment of pancreatic cancer differ slightly (152). In TCR-T, the T cells are first collected from patients; they are then selected and the TCR sequence is genetically modified to specifically bind to pancreatic cancer cells. The genetically modified T cells are then reinfused to the patient, and they attack the antigen-expressing tumor cells. In contrast, CAR-T therapy involves the introduction of a CAR into the patient’s T cells to ensure that they directly recognize and bind to pancreatic cancer cells. The genetically modified CAR-T cells are then reinfused, and they seek and destroy cancer cells that express the antigen recognized by the CAR (153, 154). Both therapies provide individualized treatment options for patients with pancreatic cancer by specifically attacking cancer cells via immune system activation.

A new immunotherapy modality used in a recently published report has gained widespread attention. This therapeutic approach has attracted considerable interest as it involves *KRAS* mutations (155), which are found in approximately 90% of patients with pancreatic cancer. The most common subtype, namely, KRAS G12D, is found in 41% of patients (156, 157). In the study (158), a patient with progressive metastatic pancreatic cancer received a single dose of genetically engineered therapy with autologous T cells which carried two allogeneic human leukocyte antigen-C*08:02-restricted TCRs directed against KRAS G12D mutations; the infusion dose was 16.2 × 10^9^ cells. Clinical evaluation at 1 month after treatment showed a reduction in the volume of visceral metastasis by 62%. Over the next 6 months, the volume of visceral metastases continued to shrink by up to 72%, and this volume remained stable 6 months later. The findings of this study offer new hope for the treatment of patients with pancreatic cancer. However, a second patient with the same *KRAS* mutation and human leukocyte antigen allele did not benefit from TCR-T cell therapy. Although the reason for the lack of benefit remains unclear, treatment-related toxicities are a concern with TCR-T therapy (150, 152). Its efficacy and the TME requirements warrant further evaluation. Despite the limitations, the case report highlights the potential efficacy of TCR gene therapy in targeting the KRAS G12D hot-spot mutation and inducing regression of metastatic pancreatic cancer, and therefore provides an important basis for future studies. In this context, TCR-T cell immunotherapy has offered remarkable results in the treatment of certain solid tumors [especially in liver cancer (159), melanoma (160), and synovial cell sarcoma (161)], where clinical data have shown strong anticancer activity.

For CAR-T therapy, the known potential antigen targets include mesothelin (162), claudin18.2 (CLDN18.2) (163), chemokine receptor 6 (CXCR6) (164), prostate stem cell antigen (PSCA) (165), carcinoembryonic antigen, and human epidermal growth factor receptor 2, among others. However, the clinical use of T-cell products targeting human epidermal growth factor receptor 2 and carcinoembryonic antigen has been associated with serious adverse events (166, 167). This limits the use of these antigens as targets for CAR-T therapy in PDAC. Conversely, autologous mesothelin-specific CAR-T cells modified using slow virus transduction have been demonstrated to be safe and have shown potential anti-tumor activity (168). In a phase III clinical trial (169) on patients with advanced pancreatic cancer that expressed glypican-1 or mesothelin, anti-mesothelin-7*19 CAR-T therapy resulted in near complete tumor eradication at 240 days after intravenous infusion. These results further suggest that the introduction of 7*19 into CAR-T cells may significantly enhance the antitumor activity against pancreatic cancer. A subtype of tight junction protein CLDN18, namely, CLDN18.2, is another potential target for CAR-T therapy. Under normal conditions, it is mainly expressed by differentiated epithelial cells of the gastric mucosa, and is expressed in up to 70% cases of gastric adenocarcinoma (170). However, CLDN18.2 activation has also been observed in pancreatic (171), esophageal (172), ovarian (173), and lung cancers (174), among others. In the most recent study (163), two patients with metastatic pancreatic cancer were treated with CLDN18.2 CAR-T after failure of standard therapy. The peripheral blood counts of CD8^+^T and regulatory T cells had increased after treatment, while those of CD4^+^T and B cells had decreased. This suggested that CAR-T therapy (targeting CLDN18.2) achieved complete remission of lung metastases and good tumor control. It further suggested that CLDN18.2 may represent a promising CAR-T target in the treatment of pancreatic cancer. In addition, CAR-T cells targeting pancreatic cancer can enhance their invasion, adhesion and therapeutic effect by overexpressing CXCR6. One study showed that engineering T cells to highly express CXCR6 made these T cells attracted to the CXC chemokine ligand 16 released by pancreatic cancer cells, resulting in enhanced T cell recognition and clearance of pancreatic cancer cells (175). This study was validated in multiple animal models, showing that CXCR6-engineered T cells were effective in tumor recognition and elimination and significantly prolonged the survival of the mice. The results of this study suggested that T cells armed with tumor-specific chemokine receptors may be an effective strategy for the treatment of pancreatic cancer and deserve further in-depth investigation.

The levels of PSCA expression have also been found to be significantly increased in primary PDAC compared to those in normal or adjacent tissues. In their study, Teng et al. (165)evaluated a novel immunotherapy modality based on human NK cells. The study evaluated the safety and efficacy of PSCA CAR NK cells that co-expressed soluble interleukin-15, with the goal of improving therapeutic efficacy. The treatment demonstrated significant tumor inhibitory effect on PSCA(+) pancreatic cancer cells *in vitro* before and after one freeze-thaw cycle. In addition to significant efficacy in the human metastatic pancreatic cancer model, the treatment demonstrated no toxic side effects. This provides a strong theoretical basis for the development of CAR-NK-based immune cell therapy for pancreatic cancer and lays the foundation for future clinical application.

Despite the favorable results, both TCR-T and CAR-T therapies are associated with a series of challenges. TCR-T therapy is associated with issues related to selection of the appropriate TCR target, affinity, and optimization, and requires the infusion of a large number of cells. CAR-T therapies are often subject to “on-target, out-of-tumor” effects, as tumor-associated antigens are present in normal tissues. In order to resolve this issue, messenger ribonucleic acid engineering methods have been used to improve CAR-T specificity (176); however, the issue pertaining to selection of therapeutic targets has not yet been addressed. Future research needs to address the limitations of these therapies and find safer and more effective therapeutic targets.

### 4.2 Oncolytic virotherapy

OVs are a class of viruses that are capable of selectively infecting and killing tumor cells without damaging normal cells (177); these include both natural and genetically engineered strains. Upon reaching the tumor site, OVs do not infect or replicate in normal cells but specifically infect tumor cells (178). They lyse the tumor cells and release progeny viruses that infect the surrounding tumor cells (179). In addition, the expression of viral antigens induces an antiviral immune response (180), which further helps destroy tumor cells. OV therapy confers the advantages of high specificity, low toxicity, and low drug resistance for the treatment of malignant tumors, especially pancreatic cancer. This treatment triggers an inflammatory cascade that simultaneously stimulates an adaptive immune response. In this context, an OVs based on modified herpes simplex virus type I, namely, talimogene laherparepvec (181) has been approved by the FDA for the treatment of melanoma. This has triggered further research on the use of OVs for the treatment of malignant tumors. The OVs currently used for the treatment of pancreatic cancer mainly include adenoviruses, reoviruses, and herpes simplex viruses, among others. These have been discussed in detail in the subsequent sections.

Adenoviruses can be engineered to carry specific genes (182) that may help inhibit tumor growth, induce apoptosis, or enhance the response of the immune system. Although meso-CAR-T has shown some efficacy in the treatment of pancreatic cancer, it has limited anti-tumor effect. In order to improve its efficacy, Watanabe et al. (183) combined meso-CAR-T cells with an oncolytic adenovirus that expressed tumor necrosis factor-α and interleukin-2. This significantly enhanced the anti-tumor effect of meso-CAR-T cells in human-PDAC-xenograft immunodeficient mice and syngeneic mouse tumor models. This enhancement was associated with a marked increase in tumor-infiltrating lymphocytes and improved T-cell function. The findings suggest that the enhancement of CAR-T cell therapy efficacy (in PDAC) using cytokine-armed oncolytic adenoviruses represents a potential strategy for overcoming the challenges associated with an immunosuppressive TME.

As the treatment of locally advanced pancreatic cancer is a particular challenge, Lee et al. (184) performed a preliminary study to evaluate the use of endoscopic ultrasound-guided injection of an adenovirus-mediated double suicide gene (Ad5-yCD/mutTK(SR39)rep-ADP) in combination with chemotherapy. Among the 11 patients who were enrolled in the study, 9 completed the assessment. One of them demonstrated a partial response at 12 weeks, and 8 had stable disease. However, adenovirus deoxyribonucleic acid was detected in the serum of 4 patients at 8 weeks (median: 55 days). These findings suggest that the combination of intratumoral replication-competent adenovirus-mediated double suicide gene therapy and gemcitabine is safe and well tolerated in patients with locally advanced pancreatic cancer. However, in-depth evaluation is needed in larger clinical trials to further confirm its effect.

PDAC is characterized by a dense connective tissue proliferative matrix (185) which limits the delivery of anticancer drugs. In this context, the oncolytic adenovirus, VCN-01, has been designed to replicate and express hyaluronidase in cancer cells with a dysfunctional RB1 pathway (186). Evaluation of its mechanism of action in preclinical models and patients with pancreatic cancer showed that combination with chemotherapy significantly improved its antitumor effect. In addition, the serum levels of hyaluronidase had increased and tumor hardness had decreased, clearly indicating the destructive effect of the adenovirus on the matrix. This provides a basis for the use of VCN-01 as a new therapeutic agent for pancreatic cancer.

Reoviruses have demonstrated potential therapeutic effects in pancreatic cancer via multiple mechanisms including oncolytic and immune activation. In a phase II trial (187) which employed reoviruses with gemcitabine for treating patients with advanced pancreatic cancer, the combination conferred a significant survival advantage. The median survival duration in the combination therapy group reached 10.2 months, and survival rates of 45% and 24% were observed at 1 and 2 years, respectively. The efficacy was notably superior to that of monotherapy with gemcitabine, and were consistent with the results obtained with the FOLFIRINOX regimen. Given the higher incidence of adverse events associated with the FOLFIRINOX regimen, these findings suggest that reoviruses plus gemcitabine may offer a superior option for the treatment of pancreatic cancer.

HF10 is a spontaneously mutated OV derived from herpes simplex virus-1 (188), that potentially exhibits potent effects against malignant tumors without damaging normal tissues. In a study (189) that included patients with unresectable advanced pancreatic cancer, substantial CD4^+^ and CD8^+^ T-cell infiltration was observed at 2 weeks after intratumoral injection of HF10; the findings suggested that this therapy may have stimulated an antitumor immune response. In another phase I clinical trial (190) from Japan, endoscopic ultrasound-guided intratumoral injection of HF10 combined with intravenous erlotinib and gemcitabine demonstrated better therapeutic efficacy than chemotherapy drugs. These findings further suggest that HF10 could be a potential therapeutic strategy for pancreatic cancer, as it stimulates the immune response and improves therapeutic efficacy. However, the deep-seated anatomical location of the pancreas renders it difficult to administer local injections. In addition, the dense stroma (191) in pancreatic cancer tissue hinders the dissemination of OVs between cancer cells. These factors limit the implementation of strategies involving local injections of OVs. Further research is therefore needed on the biological behavior of pancreatic cancer. Future studies also need to focus on the design of effective OV treatment regimens for this disease.

In conclusion, although oncolytic virotherapy has shown some potential in the treatment of pancreatic cancer, we still need to better understand a series of safety issues that may be caused by oncolytic virotherapy in clinical trials. These include the inflammatory response that can occur following viral infection, such as fever, fatigue and muscle pain. In addition, viral therapy may also lead to adverse events related to viral replication, such as abnormal liver function and hematological abnormalities. In order to solve these problems, we need to closely monitor the vital signs and laboratory indicators of patients in clinical trials, and timely detect and manage possible adverse reactions. It is worth noting that during the treatment process, OV may have an impact on normal tissues, triggering potential off-target effects, such as non-specific immune responses. Therefore, monitoring of virus spread closely during treatment is needed to assess the safety and efficacy of treatment.

### 4.3 Matrix-depletion therapy

The stroma in pancreatic cancer tissue plays an important role in tumor development; it also forms a physical barrier that limits the efficacy of treatment (192, 193). Matrix depletion therapy represents a new strategy for overcoming this challenge in the treatment of pancreatic cancer. The core goal of this therapy is to reduce the fibrous structure around the tumor and destroy the protective barrier offered by the matrix (194); this improves permeability to drugs and immune cells, thereby enhancing the therapeutic effect.

As one of the main components of the extracellular matrix, hyaluronic acid may be associated with the occurrence and development of diseases and even drug resistance. A randomized phase II trial (195) evaluated a combination of pegvorhyaluronidase alfa (PEGPH20) and nab-paclitaxel/gemcitabine versus nab-paclitaxel/gemcitabine alone in the treatment of metastatic pancreatic cancer. The results showed that patients in the combined group demonstrated higher PFS and objective response rates; in addition, the treatment effect was more obvious in patients with high levels of hyaluronic acid. In their subsequent randomized phase III trial, Van Cutsem et al. (196) evaluated the efficacy and safety of combined PEGPH20 and nab-paclitaxel/gemcitabine in patients with hyaluronic acid-high mPDAC. The results showed that although the addition of PEGPH20 increased the objective response rate, it did not improve OS or PFS. Although the benefits offered by the combination were in agreement with previous results, they did not support the use of PEGPH20 in patients with mPDAC. Further studies are required to evaluate its potential utility in these cases.

Pancreatic stellate cells play a key role in the stroma of PDAC. On activation by pro-fibrotic mediators such as transforming growth factor-β, they secrete excessive amounts of extracellular matrix proteins which produce a dense stroma (197). This in turn increases tissue fluid pressure and severely impedes diffusion of drugs from the blood vessels to the tumor tissue. Han et al. (198) therefore evaluated a two-step sequential dosing strategy for gemcitabine-based targeted therapy in pancreatic cancer. In their strategy, they employed metformin to disrupt the dense matrix; this facilitated the delivery of composite magnetic nanoparticles of gemcitabine and pH-low insertion peptide. The pH-low insertion peptide considerably enhanced the binding affinity of the nanomedicine to PANC-1 cells. The results further showed significant inhibition of tumor growth, at a rate of 91.2% after 30 days of treatment. In addition to providing an effective strategy for improving delivery efficiency of conventional drugs to the tumor site, this study expands the application of metformin to the treatment of stroma-rich malignancies.

In conclusion, stromal depletion therapy is expected to overcome the challenges posed by the stromal barrier and enhance therapeutic effects in pancreatic cancer. However, this modality is associated with multiple challenges including the physiological burden, uncertainty of treatment responses, and tolerability. Although it represents a new concept for the treatment of malignant tumors including pancreatic cancer, further studies are needed to make it suitable for clinical application. In particular, studies need to comprehensively evaluate its advantages and disadvantages in order to improve treatment safety and efficacy.

## 5 New immunotherapies

The field of innovative immunotherapy has witnessed significant advancements in pancreatic cancer research, with the identification of various potential biomarkers. These biomarkers have refined the precision of diagnosis and treatment, enabled early detection of the disease, facilitated the assessment of treatment responses, and aided in predicting prognoses. Moreover, novel combinations of immunotherapy, including the integration of endoscopy-mediated radiofrequency ablation with immunotherapy and irreversible electroporation therapy, offer more tailored and comprehensive treatment alternatives for individuals suffering from pancreatic cancer. These developments are poised to revolutionize medical practices, offering more effective and hopeful treatment avenues for patients with pancreatic cancer.

### 5.1 New potential biomarkers

Investigations into myeloid-derived suppressor cells (MDSCs) have highlighted their pivotal role in modulating immune responses, potentially influencing the effectiveness of immune cells in combating cancer. Concurrently, abnormalities in arginine metabolism have been associated with the immunotherapeutic approaches to pancreatic cancer, suggesting that further investigation could yield more effective treatment methodologies. Moreover, the role of leukemia inhibitory factor offers a novel insight, where understanding its mechanism may facilitate the development of targeted immunotherapies. These emerging biomarkers are pivotal in the realm of pancreatic cancer immunotherapy, and their interactions with the disease is extensively elucidated.

MDSCs are a heterogeneous group of bone marrow-derived cells, known for their immunosuppressive capabilities (199, 200). Normally, bone marrow cells differentiate into various immune cells, such as granulocytes, monocyte-macrophages, or dendritic cells. However, in the presence of inflammation, tumors, trauma, or other pathological states, this differentiation is halted, leading to the formation of MDSCs (201). These cells are categorized into two primary types in both humans and mice, based on their lineage: granulocytic/polymorphonuclear MDSCs (PMN-MDSCs) and monocytic MDSCs (M-MDSCs) (202). Characterized by their dampening effect on immune responses (203), MDSCs emerge as crucial contributors to tumor immune evasion. As tumors progress, an increase in MDSCs significantly influences tumor growth, metastasis, and resistance to treatment. The mounting evidence positions MDSCs not merely as a hallmark of malignancy but also as a viable target in pancreatic cancer therapy (204).

Recent studies have elucidated that exosomes released by tumor cells play a pivotal role in arresting and differentiating bone marrow hematopoietic stem cells. Macrophage migration inhibitory factor (MIF), a crucial component of tumor exosomes, emerges as a vital molecule in the induction of MDSCs (205). Research has unveiled MIF’s capacity within pancreatic cancer exosomes to trigger the differentiation of monocytes into MDSCs. IPG1576, a potent small molecule inhibitor targeting MIF tautomerase specifically, has been shown to thwart MDSC formation in the tumor microenvironment effectively, thereby enhancing CD8^+^ T cell activity and markedly reducing pancreatic cancer growth (206). This investigation posits MIF tautomerase inhibitors as promising agents in pancreatic cancer therapy, heralding a novel tactic for treating such tumors. The potential of small molecule drugs, engineered through MIF tautomerase, shines a new light on future cancer treatments. Furthermore, the characteristic immunosuppression associated with tumor progression can be countered by inhibiting or depleting M-MDSCs, thus fostering antitumor immunity. Researchers found that a significant correlation between the presence of chemokine receptor 2 (CCR2) and MDSCs in tumor tissues and the survival rates of pancreatic cancer patients has been observed in surgical specimens (207). Utilizing a CCR2 blocker, PF-04136309, within an orthotopic mouse model of pancreatic cancer demonstrated the ability to decelerate tumor growth and metastasis by diminishing MDSC infiltration in tumor tissues via the CCL2/CCR2 pathway (208). The CCL2/CCR2 chemokine axis is known to facilitate the migration of M-MDSCs to tumor sites, cultivating an immunosuppressive microenvironment. This pathway holds prognostic value in pancreatic cancer, where blocking CCR2 can rejuvenate anti-tumor immunity. These findings suggest a viable therapeutic strategy in managing MDSC invasion through the CCL2/CCR2 pathway, offering an enhanced immunotherapy approach for pancreatic cancer patients.

Arginine, a conditionally non-essential amino acid, plays a pivotal role in the metabolism of various malignant tumors, including pancreatic cancer (209). The metabolism alteration in these cancers is primarily evidenced by the suppression of argininosuccinate synthase 1 (ASS1), a crucial enzyme in arginine synthesis (210). Consequently, treatments that deplete arginine levels can markedly impair the proliferation, invasion, and migration of pancreatic cancer cells, characterized by reduced ASS1 activity (211). Arginine-deprivation therapies, such as ADI-PEG 20—a pegylated form of arginine deiminase—specifically target pancreatic cancer cells deficient in ASS1. In an advanced pancreatic cancer phase 1/1B single-arm clinical trial (212), a regimen combining ADI-PEG20, gemcitabine, and nab-paclitaxel demonstrated tolerability and efficacy in both previously treated and untreated advanced pancreatic cancer patients, including those with ASS1-deficient tumors. The regimen involved administering gemcitabine (1000 mg/m^2^) and nab-paclitaxel (125 mg/m^2^) for 3 weeks, along with a weekly intramuscular injection of ADI-PEG 20 at 36 mg/m^2^. The observed overall response rate was 45.5% (5 out of 11 patients), with a median PFS of 6.1 months (95% CI, 5.3-11.2 months). Treatment-related adverse effects included neutropenia, thrombocytopenia, leukopenia, anemia, peripheral neuropathy, and fatigue. These findings necessitate further investigation to establish ASS1 expression as a predictive biomarker for arginine deprivation therapy efficacy. Nonetheless, arginine deprivation presents a promising therapeutic approach for future treatment modalities in pancreatic cancer patients.

Leukemia inhibitory factor (LIF), a member of the interleukin-6 cytokine family, mediates signal transduction between pancreatic cancer cells and stellate cells, emerging as a pivotal factor in the regulation of cell differentiation, renewal, and survival. Its role in supporting cancer progression positions LIF as a potential therapeutic target for pancreatic cancer (213). Research by Shi et al. (214)has shown that both the pharmacological inhibition of LIF and the genetic deletion of LIF markedly delay tumor progression and augment chemotherapy effectiveness by altering cancer cell differentiation and the epithelial-mesenchymal transition status, consequently extending survival in a mouse model of PDAC. Additionally, aberrant LIF production in the pancreas is observed exclusively under pathological conditions in both mouse models and human PDAC, linking it to the disease’s pathogenesis. Collectively, these findings underscore LIF’s critical role in PDAC development and its potential as a therapeutic target and biomarker, meriting further clinical exploration.

In recent years, the imbalance of intestinal flora has become an important link in the occurrence and development of pancreatic cancer (215). Intestinal microbiota and microbial metabolites play an important role in chemotherapy and immunotherapy of pancreatic cancer. One study found that indole-3-acetic acid, a tryptophan metabolite produced by two gut bacteria (bacteroides fragilis, bacteroides polymorpha), was found to be at better levels in chemotherapeutic PDAC patients, and chemotherapy efficacy could be enhanced by direct indole-3-acetic acid supplementation or by performing a high-tryptophan diet (216). This provides the impetus to consider nutritional interventions during the treatment of cancer patients. In addition, the intratumoral and gut microbiota can have significant effects on innate and adaptive immunity, and therefore can also determine cancer progression and response to therapy in part. Gut microbiota-derived trimethylamine *N*-oxide has been shown to have immunomodulatory effects and thus may be a therapeutic entry point to enhance anti-tumor immune responses, thus enabling PDAC to respond to checkpoint immunotherapy (217). This study lays a foundation for potential therapeutic strategies targeting trimethylamine *N*-oxide and provides a new idea for the treatment of pancreatic cancer.

In summary, investigating these biomarkers’ interplay offers deeper insights into the patient’s immune system status, enabling more precise, personalized treatment plans and advancing the application of immunotherapy in pancreatic cancer treatment. Such research illuminates new avenues for future clinical practice and innovative strategies to navigate the complexities of pancreatic cancer therapy.

### 5.2 New potential immunotherapeutic combinations

Recently, there have seen considerable innovation in pancreatic cancer immunotherapy, introducing numerous promising therapeutic combinations. These include endoscopy-mediated radiofrequency ablation (RFA), immunotherapy, and irreversible electroporation therapy, as well as the integration of selective multikinase inhibitors and immunotherapy. Such combinations unveil new avenues for clinical application. RFA offers a minimally invasive method to eliminate cancer lesions, setting the stage for further treatments (218). Immunotherapy, on the other hand, stimulates the immune system, intensifying its response to pancreatic cancer cells. Irreversible electroporation therapy enhances treatment efficacy via the electric field effect (219), whereas the synergy of selective multikinase inhibitors with immunotherapy transforms the tumor microenvironment, increasing the susceptibility of pancreatic cancer to treatment (220). The detailed application of these three combination therapies in the clinical management of pancreatic cancer will be elaborated upon below.

RFA has gained recognition as an innovative approach for treating locally advanced, unresectable pancreatic cancer (LAPC). A phase II study aimed to assess the safety of RFA in LAPC patients (221). The findings indicate that RFA constitutes a viable and safe surgical intervention for LAPC when applied in strict adherence to established safety protocols. Further research involving endoscopic ultrasonography-guided RFA of pancreatic neuroendocrine and cystic tumors has confirmed its safety (222), reporting a complication rate of 10%. Enhancing surgical precautionary measures could mitigate the risk of complications. Additionally, the integration of irreversible electroporation with immunotherapy (nivolumab) in a phase 1b trial for LAPC demonstrated tolerability without dose-limiting toxicities (223). A multicenter, phase 2 adjuvant trial combining irreversible electroporation and nivolumab is currently underway for patients with LAPC, showing potential therapeutic benefits and warranting further exploration. Intriguingly, Falcomatà et al. (224)discovered that selective multikinase inhibitors rendered mesenchymal pancreatic cancer more amenable to immune checkpoint blockade by altering the tumor microenvironment. A comprehensive drug screen of 418 compounds on human PDAC and mouse cancer cells revealed that trametinib and nintedanib combination therapy inhibited the cell cycle, induced cancer cell death, and enhanced the tumor microenvironment for more effective T cell activity. The combination of immunotherapy (anti-PD-L1 inhibitor) with trametinib and nintedanib markedly improved treatment responses in mice, with the triple therapy group achieving a significant survival benefit over controls. This research paves the way for novel treatment strategies targeting the challenging and resistant stromal subtype of PDAC.

In summary, these multi-faceted combined treatment strategies offer comprehensive and personalized therapeutic options, presenting new hope and promising prospects for individuals with pancreatic cancer. This approach establishes a robust foundation for future clinical applications. However, it is imperative to closely monitor potential adverse reactions to optimize their application in patient care, ensuring these combined treatments are administered with enhanced safety and efficacy for pancreatic cancer patients.

## 6 Conclusion and future perspectives

PDAC is a highly heterogeneous malignant tumor with a high postoperative recurrence rate. As traditional treatment methods are largely ineffective, patients with PDAC have a poor prognosis. Although traditional immunotherapy has been effective in treating a wide variety of malignant tumors in recent years, the inherent genetic instability, local immunosuppressive microenvironment, and barrier effect of the dense matrix considerably impair its therapeutic effect in pancreatic cancer. Emerging tumor immunotherapy approaches provide new hope for the treatment of pancreatic cancer. The development of ICIs is an important breakthrough in PDAC, and its antitumor effects have been demonstrated in patients. However, concerns pertaining to their toxicity and efficacy largely limit widespread clinical application. Cancer vaccines, ACT, OVs, and matrix modulators have been demonstrated to offer potential benefits in the treatment of pancreatic cancer, they are associated with a series of challenges. For instance, further research is needed to address the issues of tumor heterogeneity and individual differences in relation to cancer vaccines, uncertainty of treatment effect with ACT, and the need to pay attention to its toxicity. OVs demonstrate good tumor specificity, their safety and efficacy warrants further validation, and the issue of antibody neutralization needs to be resolved. Matrix modulators have improved the microenvironment of immunosuppression in pancreatic cancer, the issues with treatment complexity and tolerability need to be addressed. In order to address the limitations of immunotherapy for PDAC, it is essential that further studies aim at obtaining an in-depth understanding of the TME in pancreatic cancer, improving the off-target effects of ACT, summarizing the reasons for the failure of immunotherapy drugs, and evaluating the feasibility of combined treatment strategies.

Future research needs to explore more specific biomarker molecules as well as immunotherapy combinations, and developing new targeted drugs and cancer vaccines to better address the multiple immune deficiencies in patients with PDAC. This may help improve the safety and efficacy of immunotherapy. Studies need to evaluate the best immunotherapy combinations (**Figure 3**), patient selection criteria, and the timing of treatment. Due to the suppressive effect of the microenvironment of pancreatic cancer on immunity, single immunotherapy is often difficult to achieve ideal efficacy (225). Therefore, the current research trend is to overcome the immunosuppression in the microenvironment of pancreatic cancer and enhance the ability of immune cells to recognize and eliminate tumors by combining a variety of immunotherapy methods or combining immunotherapy with other treatment methods (226), so as to improve the therapeutic effect.

In conclusion, it is essential to tailor treatment strategies based on the patient condition to ensure optimal patient and tumor outcomes. Focusing on the use of individualized and combined treatment strategies may effectively reduce adverse reactions and improve comprehensive treatment of pancreatic cancer. Finally, immunotherapy for pancreatic cancer is associated with both hopes and challenges. Continuous in-depth research and exploration is warranted to improve patient outcomes.

## Author contributions

RZ: Writing – original draft, Writing – review & editing, Conceptualization, Software. XL: Conceptualization, Writing – original draft, Writing – review & editing. YZ: Data curation, Methodology, Writing – original draft. YXL: Formal analysis, Supervision, Writing – original draft. YW: Formal analysis, Resources, Writing – original draft. SG: Formal analysis, Project administration, Writing – review & editing. XJ: Data curation, Formal analysis, Writing – original draft. JZ: Conceptualization, Investigation, Writing – review & editing. YG: Conceptualization, Visualization, Writing – original draft. YL: Funding acquisition, Resources, Supervision, Visualization, Writing – original draft, Writing – review & editing.
